# A Zeolitic Pyrimidine Framework (ZPF) Nanoplatform Co‐Delivers a DNAzyme and a Protein Prodrug for Cascade‐Activated Tumour Therapy

**DOI:** 10.1111/cpr.70225

**Published:** 2026-04-27

**Authors:** Yan Huang, Xingjie Hu, Jinli Sun, Min Yin, Nan Chen

**Affiliations:** ^1^ Shanghai Engineering Research Center of Green Energy Chemical Engineering, Key Laboratory of Resource Chemistry of Ministry of Education, Shanghai Frontiers Science Center of Biomimetic Catalysis, College of Chemistry and Materials Science Shanghai Normal University Shanghai China; ^2^ State Key Laboratory of Oncogenes and Related Genes, Center for Single‐Cell Omics, School of Public Health Shanghai Jiao Tong University School of Medicine Shanghai China

**Keywords:** cascade activation, DNAzyme, ROS‐responsive prodrug, synergistic therapy, targeted protein therapy, zeolitic pyrimidine framework (ZPF)

## Abstract

The therapeutic application of cytotoxic proteins like ribonuclease A (RNase A) is hindered by their systemic toxicity. Here, we engineer a protein‐structure‐directed zeolitic pyrimidine framework (ZPF) as a superior nanocarrier that co‐encapsulates and stabilizes a ROS‐responsive RNase A prodrug (RNBC) and a GPX‐1‐targeting DNAzyme with high efficiency. This DZ‐RNBC@ZPF nanocomplex, camouflaged with a 4T1 cell membrane, ensures tumour‐specific delivery and lysosomal degradation. The acidic environment triggers framework dissolution, releasing Zn^2+^ to activate the DNAzyme. The DNAzyme downregulates GPX‐1, amplifying intracellular H_2_O_2_, which in turn cleaves the NBC group to activate the RNase A prodrug precisely within target cells. This coordinated intracellular cascade—from material degradation to enzymatic ROS amplification and prodrug activation—induces significant apoptosis in 4T1 cells and potently suppresses tumour growth in vivo. Our work establishes ZPF as an enabling platform for the co‐delivery of bioactive macromolecules and presents a novel therapeutic strategy based on a synergistic protein prodrug‐DNAzyme circuit.

## Graphical abstract

A zeolitic framework co‐delivers a DNAzyme and a protein prodrug to tumour cells and executes a self‐amplifying therapeutic cascade: the DNAzyme elevates tumour oxidative stress to activate a caged RNase A prodrug. This spatiotemporally controlled synergy converts a genetic input into potent enzymatic cytotoxicity, suppressing tumour growth.

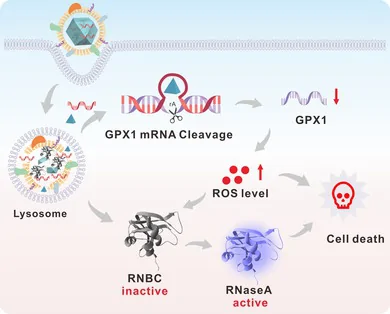

## 1 Introduction

Protein‐based therapeutics represent a cornerstone of modern molecular medicine due to their high specificity and potent biological functions [1, 2, 3]. However, translating the anti‐cancer potential of cytotoxic proteins into clinical success is frequently thwarted by two formidable barriers: the lack of efficient intracellular delivery and the absence of tumour‐specific activation triggers [4, 5, 6, 7]. To mitigate off‐target toxicity, therapeutic proteins are often “caged” or modified into pro‐enzymes that require specific stimuli for reactivation [8, 9, 10, 11]. Among available triggers, reactive oxygen species (ROS) have emerged as promising candidates, capitalizing on the intrinsically high oxidative stress of the tumour microenvironment (TME) [12, 13, 14, 15, 16, 17]. Yet, endogenous ROS levels in many malignancies often fall below the threshold required for efficient prodrug conversion, leading to suboptimal therapeutic effects [18, 19].

To overcome this ‘trigger insufficiency’, current strategies often rely on external energy inputs—such as light or ultrasound—which are limited by tissue penetration depth and complex clinical setups [20, 21, 22]. A more elegant, autonomous solution lies in intracellular cascade biocatalysis, where the nanoplatform itself orchestrates a series of biochemical events to amplify endogenous signals [23, 24, 25]. DNAzymes (DZs), which are single‐stranded catalytic DNA molecules, offer a programmable means of gene regulation [26, 27, 28, 29, 30]; however, their intracellular performance is often hampered by the scarcity of essential metal‐ion cofactors and poor endosomal escape [31, 32, 33]. Integrating the genetic precision of DZs with the cytotoxic potency of protein prodrugs into a single, self‐amplifying feedback loop remains a major frontier in intelligent nanomedicine.

In this study, we present a cascade‐activated biocatalytic nanoplatform, DZ‐RNBC@ZPF, designed to translate a genetic “input” into an amplified enzymatic “output” via a redox‐driven feedback loop. We utilize a Zeolitic Pyrimidine Framework (ZPF) to co‐encapsulate a GPX1‐targeting DZ and a ROS‐responsive RNase A prodrug (RNBC) [34, 35, 36]. The ZPF matrix serves a dual role: it provides structural stabilization for the biomacromolecules and acts as an intracellular reservoir for Zn^2+^ cofactors. Upon acidic disassembly in the lysosome, the released Zn^2+^ activates the DZ to silence GPX1 mRNA, thereby paralysing the cell's antioxidant defences and triggering a ROS burst. This amplified oxidative environment subsequently cleaves the boronate linkages of the RNBC, restoring the catalytic activity of RNase A to induce systemic RNA degradation and apoptosis.

By coupling gene regulation with protein biochemistry, our platform establishes a self‐reinforcing therapeutic circuit that operates with high spatial fidelity and minimal off‐target effects. This work provides a generalizable blueprint for the design of autonomous, signal‐transducing nanomachines capable of processing complex endogenous cues for precision cancer therapy (Scheme 1).

**SCHEME 1:**
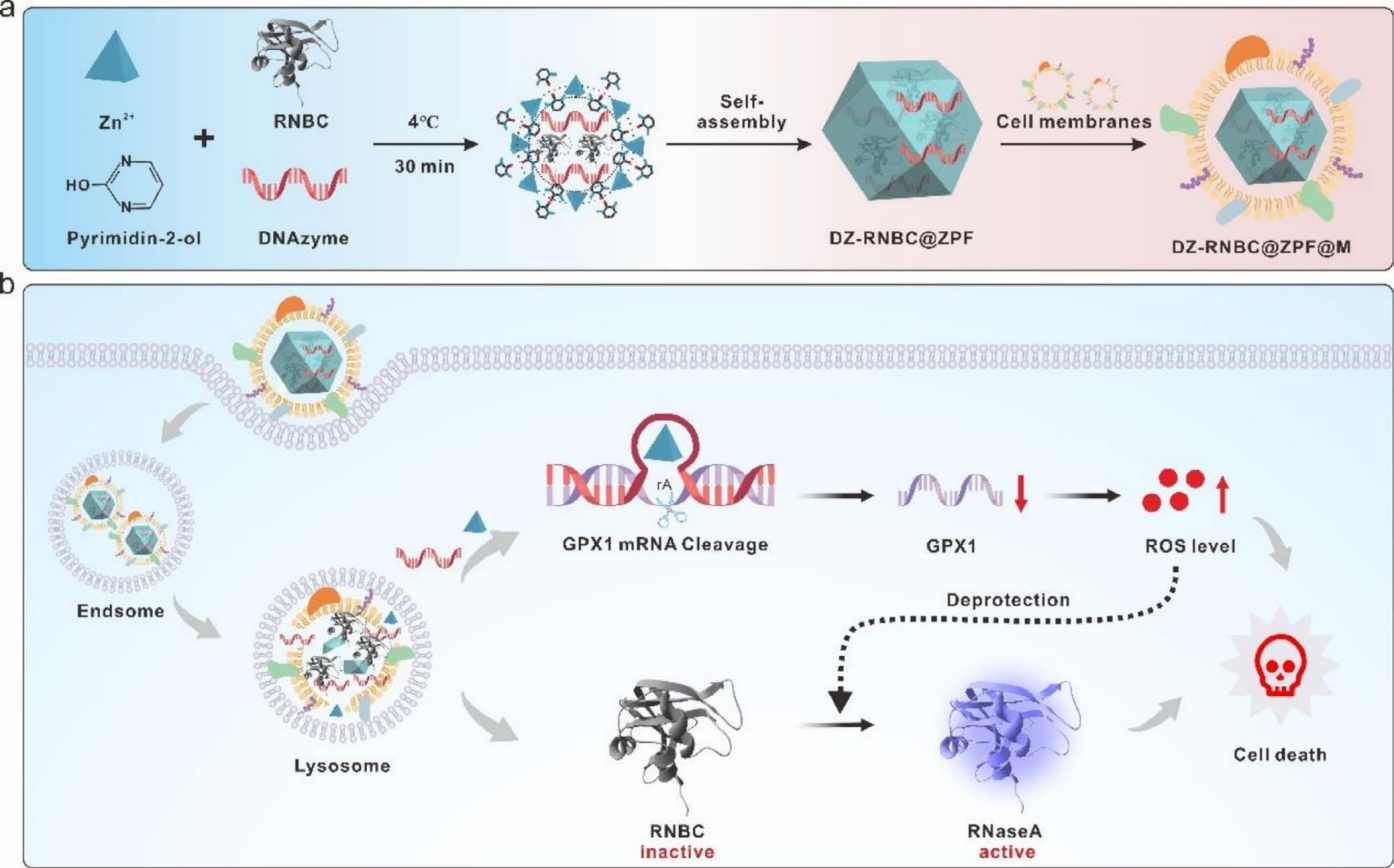
Bioresponsive nanoplatform for redox‐amplified cancer therapy. (a) One‐pot biomimetic synthesis of the zeolitic pyrimidine framework (ZPF) co‐encapsulating a GPX1‐targeting DNAzyme (DZ) and a ROS‐responsive RNase A prodrug (RNBC), followed by 4T1 tumour cell membrane coating (M) for targeted delivery. (b) Therapeutic cascade: (i) Membrane‐mediated cellular internalization and lysosomal acid‐triggered ZPF degradation release Zn^2+^ and cargo; (ii) Zn^2+^ activates DZ to cleave GPX1 mRNA, suppressing cellular antioxidant defences and amplifying ROS; (iii) Elevated ROS cleaves the RNBC prodrug, activating RNase A to degrade RNA and induce apoptosis.

## 2 Materials and Methods

### 2.1 Materials and Reagents

RNase A and the ROS probe 2′,7′‐dichlorofluorescin diacetate (DCFH‐DA) were purchased from Sigma‐Aldrich. The GPX1‐targeting DZ (sequence: 5′‐GATCGTGGTGCTCCGAGCCGGTCGAACAGAGAGACGCG‐3′) and its fluorescently labelled variants (Cy3‐labelled) were synthesized by Sangon Biotech (Shanghai, China). 4‐(Hydroxymethyl)phenylboronic acid pinacol ester, p‐nitrophenyl chloroformate, and 2‐hydroxypyrimidine were obtained from Shanghai Bide Pharmaceutical. Zinc nitrate hexahydrate was sourced from the Shanghai Discovery Platform. All other chemicals were of analytical grade and used without further purification. Deionized water (18.2 MΩ cm) was used for all aqueous solution preparations.

### 2.2 Synthesis of the ROS‐Responsive RNBC Prodrug

#### 2.2.1 Synthesis of the NBC Linker

The ROS‐cleavable linker, 4‐nitrophenyl 4‐(4,4,5,5‐tetramethyl‐1,3,2‐dioxaborolan‐2‐yl) benzyl carbonate (NBC), was synthesized by reacting 4‐(hydroxymethyl)phenylboronic acid pinacol ester (2.1 mmol) with p‐nitrophenyl chloroformate (2.3 mmol) in anhydrous dichloromethane (DCM, 10 mL) containing triethylamine (4.3 mmol). The reaction proceeded at room temperature overnight. The product was purified via silica gel column chromatography [37, 38].

#### 2.2.2 Conjugation to RNase A

To prepare the proenzyme RNBC, native RNase A (6 mg) was dissolved in NaHCO₃ buffer (0.1 M, pH 8.5, 2 mL). An NBC solution (9.6 mg in 300 μL DMSO) was added dropwise. After reacting for 12 h at room temperature with gentle stirring, the mixture was dialyzed against deionized water (MWCO: 3.5 kDa) for 24 h and lyophilized. The modification degree was quantified using MALDI‐TOF mass spectrometry.

### 2.3 Fluorometric Alizarin Red S Assay for Boronate Verification

An Alizarin Red S (ARS) assay was performed to confirm the presence of the boronic acid moiety in RNBC. Protein samples (0.2 mg/mL), including native RNase A, RNBC, and RNBC pre‐treated with H_2_O_2_ (100 μM, 24 h), were incubated with a 0.02% (w/v) ARS solution in PBS (pH 7.4) for 2 h at room temperature in the dark. Fluorescence intensity was measured at an emission of 600 nm with excitation at 490 nm [37].

### 2.4 Agarose Gel Electrophoresis for RNase Activity Assay

RNase activity was assessed via agarose gel electrophoresis. RNBC (0.25 mg/mL) was first treated with or without H_2_O_2_ (100 μM) for 24 h. Subsequently, the treated RNBC, untreated RNBC, and native RNase A (each at 0.15 μg/mL final protein concentration) were separately incubated with RNA (0.25 mg/mL) in Tris‐HCl buffer (10 mM, pH 7.5) for 15 min at 37°C. The reactions were stopped by adding loading dye, and the mixtures were analysed on a 1.5% agarose gel stained with GelRed.

### 2.5 Fabrication and Characterization of the Nanoplatform

#### 2.5.1 Synthesis of DZ‐RNBC@ZPF

The DZ‐RNBC@ZPF nanocomplex was constructed via a one‐pot biomimetic mineralization strategy. Briefly, an aqueous solution containing RNBC (2 mg) and the GPX1‐targeting DZ (0.1 mg) was mixed with Zn(NO_3_)_2_·6H_2_O (20 mg in 1 mL H_2_O). Subsequently, an aqueous solution of 2‐hydroxypyrimidine (19 mg) was added to initiate coordination‐driven self‐assembly. The reaction was maintained at 4°C for 30 min under vigorous stirring. The resulting precipitates were collected by centrifugation (10,000 rpm, 10 min) and washed three times with deionized water.

#### 2.5.2 Membrane Coating (DZ‐RNBC@ZPF@M)

4T1 cells cultured in dishes were harvested using a cell scraper and collected by centrifugation. Hypotonic lysis buffer containing Tris‐HCl (20 mM), KCl (10 mM), MgCl_2_ (10 mM), and EDTA (1 mM) was prepared. The cell pellet was resuspended in the hypotonic buffer and disrupted by repeated grinding with a mini‐grinder. The resulting homogenate was centrifuged at 3200 ×*g* for 5 min at 4°C to remove nuclei and intact cells. The supernatant was then collected and centrifuged at 100,000 ×*g* for 1 h at 4°C to obtain purified 4T1 cell membrane fragments.

For membrane coating, the purified 4T1 cell membranes (equivalent to 1 mg of protein) and the DZ‐RNBC@ZPF nanocomplex (2 mg) were co‐dispersed in PBS. The mixture was sonicated in an ice bath (80 W, 3 min) and then sequentially extruded through a 200 nm polycarbonate membrane using a mini‐extruder to yield the membrane‐cloaked DZ‐RNBC@ZPF@M nanoparticles.

#### 2.5.3 Determination of Drug Loading Efficiency

The loading content of DZ was determined by quantifying non‐encapsulated cargo in the supernatant after synthesis. For the fluorescently labelled DZ (Cy3‐DZ), the supernatant fluorescence was measured (Ex/Em: 550/570 nm). For RNBC quantification, DZ‐RNBC@ZPF was completely dissolved in acid, then the protein concentration was determined using the Bradford assay. The loading efficiency was calculated as follows: DLC (%) = (Weight of loaded drug/Total weight of nanocomplex) × 100%.

### 2.6 Physicochemical Characterization

The hydrodynamic particle size, polydispersity index (PDI), and zeta potential were measured using Dynamic Light Scattering (DLS) at 25°C. Morphology was characterized by Scanning Electron Microscopy (SEM).

### 2.7 In Vitro Release Kinetics Study

The pH‐responsive release of the DNAzyme from the nanocomplex was investigated. DZ‐RNBC@ZPF loaded with Cy3‐labelled DZ (0.5 mg/mL) was dispersed in 1.5 mL of release media (0.01 M PBS) at different pH values (5.0 and 7.4) and incubated at 37°C with gentle shaking. At predetermined time points, samples were centrifuged (12,000 ×*g*, 5 min). The fluorescence intensity of the supernatant was measured and compared to a standard curve of free Cy3‐DZ to calculate the cumulative release percentage.

### 2.8 Cell Culture

4T1 murine breast cancer cells (Shanghai Institute of Biological Sciences) were cultured in RPMI‐1640 medium supplemented with 10% heat‐inactivated foetal bovine serum (FBS), 100 U/mL penicillin, and 100 μg/mL streptomycin at 37°C in a humidified 5% CO_2_ incubator.

### 2.9 In Vitro Cellular Assays

#### 2.9.1 Cellular Uptake and Mechanism Study

4T1 cells were seeded in confocal dishes and incubated to 70%–80% confluence. Cells were then treated with fluorescently labelled nanoparticles (Alexa647‐RNBC@ZPF) at varying concentrations and for different durations. For inhibition studies, cells were pre‐treated with methyl‐β‐cyclodextrin (MβCD, 5 mM) or chlorpromazine (CPZ, 10 μg/mL) for 1 h, or incubated at 4°C for 30 min prior to nanoparticle exposure. Intracellular fluorescence was analysed using Confocal Laser Scanning Microscopy (CLSM).

#### 2.9.2 GPX1 mRNA Knockdown

After a 24‐h treatment with various formulations, total RNA was extracted from 4T1 cells. GPX1 mRNA levels were quantified via RT‐qPCR using GAPDH as the internal reference.

#### 2.9.3 ROS Detection

Following treatment, cells were incubated with 10 μM DCFH‐DA for 30 min at 37°C. Intracellular ROS generation was visualized via CLSM and quantified by flow cytometry.

#### 2.9.4 Lysosomal Escape and Payload Separation

4T1 cells were treated with dual‐labelled nanoparticles (Cy3‐DZ and Alexa647‐RNBC). The spatial separation of the red (Cy3) and far‐red (Alexa647) fluorescence signals within cells was tracked over time using CLSM to confirm lysosomal escape and payload dissociation.

#### 2.9.5 MTT Assay

4T1 cells were seeded in 96‐well plates and treated with various formulations for 24 h. MTT reagent (0.5 mg/mL) was added and incubated for 4 h. The formazan crystals were dissolved in DMSO, and absorbance was measured at 570 nm.

#### 2.9.6 Live/Dead Staining

Treated cells were stained with Calcein‐AM (2 μM, green for live cells) and Propidium Iodide (PI, 4 μM, red for dead cells) for 30 min and imaged using fluorescence microscopy.

### 2.10 Confocal Fluorescence Microscopy and Image Analysis

Cells were imaged using a Leica SP8 confocal microscope. Alexa 647‐labelled RNBC was excited with a 633 nm HeNe laser, Cy3‐labelled DZ with a 561 nm laser, LysoTracker Green with a 488 nm Ar‐Kr laser, and Hoechst 33258 with a 405 nm diode laser. Emission was collected at 650–670, 570–620, 500–550, and 450–500 nm, respectively. Image analysis was performed using ImageJ (FIJI) software. Colocalization analysis was conducted using the Manders' colocalization coefficients (M1 and M2), which represent the fraction of overlap and range from 0% to 100%. The Costes method was used for statistical significance testing of pixel intensity correlation (100 randomizations).

### 2.11 In Vivo Antitumor Efficacy

Female BALB/c mice (6–8 weeks) were obtained from SLAC Laboratory Animal Co. Ltd. All mouse experiments were conducted following protocols approved by the Animal Care and Use Committee of Shanghai Jiao Tong University School of Medicine (approval number: A‐2022‐115). A syngeneic orthotopic breast cancer model was established by injecting 4T1 cells (2 × 10^5^ in 50 μL PBS) into the mammary fat pad of female BALB/c mice (6–8 weeks old). When tumour volumes reached approximately 80 mm^3^, mice were randomly divided into groups (*n* = 7) and administered intravenous injections of: (1) PBS, (2) RNBC@ZPF@M, or (3) DZ‐RNBC@ZPF@M (at a dose equivalent to 5 mg/kg of RNBC). Injections were administered on days 5, 8, 11, and 14. Tumour dimensions (length and width) and body weights were monitored every 2 days. Tumour volume was calculated as: Volume = (Length × Width^2^)/2.

### 2.12 Histological and Biosafety Analysis

On day 17, mice were euthanized. Tumours and major organs (heart, liver, spleen, lungs, kidneys) were harvested, weighed, and fixed in 4% paraformaldehyde. Tumour tissues were paraffin‐embedded, sectioned, and subjected to Haematoxylin & Eosin (H&E) staining, TUNEL assay (for apoptosis), and immunohistochemical staining for Proliferating Cell Nuclear Antigen (PCNA). Major Organs were sectioned and stained with H&E to assess potential systemic toxicity. Serum Biochemistry: Blood samples were collected, and serum levels of alanine aminotransferase (ALT), aspartate aminotransferase (AST), blood urea nitrogen (BUN), and creatinine (Cr) were measured using standard commercial kits.

### 2.13 Statistical Analysis

All data are presented as mean ± standard deviation (SD). Statistical comparisons between two groups were performed using Student's unpaired two‐tailed *t*‐test. Comparisons among multiple groups were conducted using one‐way analysis of variance (ANOVA) with Tukey's post hoc test. A *p* value of less than 0.05 (*p* < 0.05) was considered statistically significant.

## 3 Results

### 3.1 Design and Molecular Engineering of the DZ‐RNBC@ZPF Nanoplatform

To overcome the “trigger insufficiency” common to many prodrug strategies, we engineered a biocatalytic system designed for self‐amplification. We first synthesized the ROS‐cleavable masking agent, 4‐nitrophenyl 4‐(4,4,5,5‐tetramethyl‐1,3,2‐dioxaborolan‐2‐yl) benzyl carbonate (NBC), via a two‐step esterification process. The structure of NBC was confirmed via ^1^H NMR, which displayed characteristic peak shifts corresponding to the arylboronate moiety (Figure S1). RNBC was then generated by covalently conjugating the NBC groups to the ε‐amino groups of lysine residues on the RNase A surface. This chemical masking is critical; lysine residues are essential for the phosphate‐binding pocket of RNase A, and their blockage effectively silences enzymatic activity (Figure 1a). MALDI‐TOF mass spectrometry provided quantitative validation of this modification, revealing a mass shift from 13,683 Da (native RNase A) to 14,608 Da (RNBC). This increase corresponds to the conjugation of approximately four NBC moieties per protein molecule, a modification density sufficient to sterically and chemically inhibit the enzyme without inducing irreversible denaturation (Figure 1b,c). SDS‐PAGE showed the expected mobility shift (Figure S2a), and zeta potential shifted from +3 to −31.8 mV, consistent with lysine masking (Figure S2b).

**FIGURE 1:**
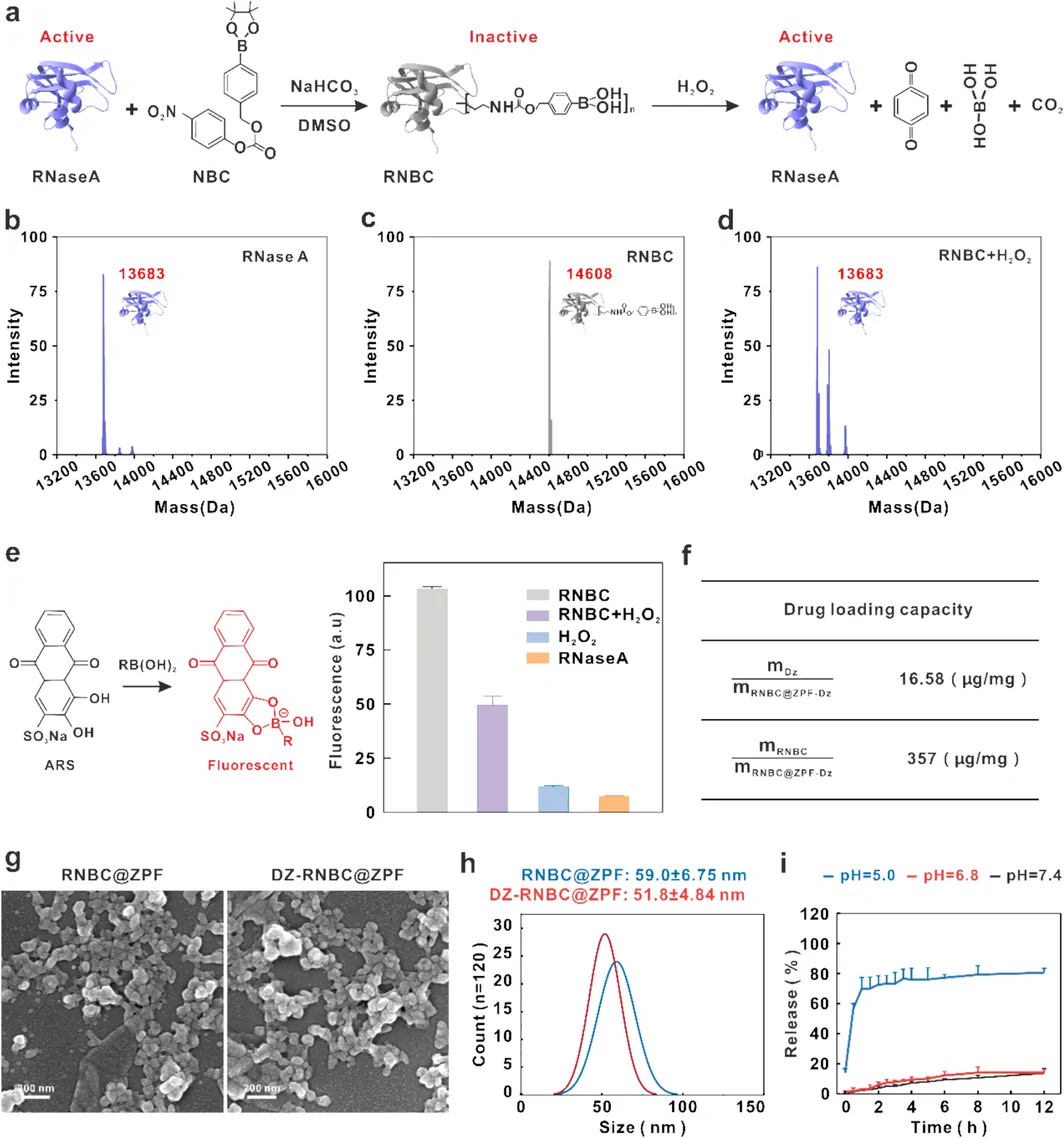
Synthesis and characterization of the RNBC prodrug and DZ‐RNBC@ZPF nanocomposite. (a) Synthetic scheme for the NBC‐modified RNase A (RNBC) with H_2_O_2_‐responsive activation mechanism. (b–d) MALDI‐TOF mass spectrometry analysis of (b) native RNase A (13.7 kDa), (c) RNBC (+4 NBC modifications), and (d) H_2_O_2_‐treated RNBC showing complete restoration of native mass, confirming reversible prodrug conjugation. (e) Fluorescence quantification of released Alizarin Red S from boronate ester cleavage, demonstrating selective RNBC activation by H_2_O_2_ compared to untreated controls. (f) Encapsulation efficiency of DZ and RNBC in ZPF. (g and h) Morphological characterization: (g) SEM micrographs and (h) diameter distribution showing uniform spherical nanoparticles. (i) pH‐responsive release profile of Cy3‐DZ from ZPF, Data represent mean ± SD (*n* = 3). Scale bars: 200 nm.

The reversibility of this “caging” strategy was verified. Upon H_2_O_2_ exposure, the arylboronate ester cleaves, restoring the native protein mass as confirmed by mass spectrometry (Figure 1d). The ROS sensitivity of the boronate linkage was further validated through an ARS displacement assay, where H_2_O_2_ treatment significantly quenched the fluorescence of the ARS–boronate complex (Figure 1e). Critically, agarose gel electrophoresis demonstrated that while RNBC was inert, H_2_O_2_‐treated RNBC regained full RNA‐degrading activity (Figure S3). However, basal cancer cell ROS levels are often insufficient for complete prodrug activation. To address this, we designed a DZ to cleave GPX1 mRNA, aiming to amplify intracellular ROS as a more potent trigger (Figure S4).

To co‐deliver these agents, we fabricated the DZ‐RNBC@ZPF nanocomplex via a one‐pot biomimetic mineralization approach. The ZPF simultaneously encapsulates RNBC and the DZ while incorporating Zn^2+^ ions into its lattice. This “one‐pot” assembly serves dual purpose: encapsulating the biologics to protect them from premature enzymatic degradation and storing Zn^2+^ in the carrier lattice. Importantly, these lattice‐bound zinc ions are not merely structural; upon framework degradation, they function as essential cofactors for the catalytic activity of the DZ, establishing a self‐supplying catalytic circuit.

The encapsulation efficiency was high, with loading capacities of 357 μg/mg for RNBC and 16.58 μg/mg for DZ (Figures 1f and S5). SEM imaging revealed monodisperse, irregular spherical nanoparticles with mean diameters of 51.8 ± 4.8 nm for DZ‐RNBC@ZPF and 59.0 ± 6.8 nm for RNBC@ZPF, an ideal size for tumour penetration (Figure 1g,h). The negative zeta potential decreased further upon DZ loading, suggesting successful DZ incorporation (Figure S6). The complexes showed excellent stability over 7 days. DLS monitoring showed negligible variations in particle size or PDI, confirming excellent colloidal stability in aqueous media (Figure S7). Stability of the framework is pH‐responsive. It is stable at physiological pH (7.4) and in the mild acidic TME (pH 6.8), whereas it disassembles under acidic conditions (pH 5.0), releasing over 80% of the DZ payload within 12 h (Figure 1i). To provide direct structural evidence for the acid‐triggered disassembly, SEM imaging of the DZ‐RNBC@ZPF nanoparticles was performed after incubation in acetate buffer at pH 5.0 for 1 h. It maintained a spherical and intact morphology at pH 7.4. In contrast, it exhibited obvious signs of dissolution after 30 min, at pH 5.0, followed by pronounced structural collapse and fragmentation after 60 min, directly confirming the disassembly of the ZPF framework under acidic conditions (Figure S8). This ensures the therapeutic cascade initiates specifically after lysosomal internalization.

### 3.2 Spatiotemporal Internalization and Intracellular Trafficking

Efficient intracellular delivery and subsequent endosomal escape are prerequisites for the function of macromolecular therapeutics. We visualized delivery using CLSM with Alexa647‐labelled RNBC. DZ‐RNBC@ZPF internalization was concentration‐ and time‐dependent, with strong fluorescence after 9 h (Figure 2a–d). Mechanistic inhibitor studies revealed that uptake was significantly attenuated at 4°C and upon pretreatment with MβCD, indicating a primary, energy‐dependent caveolin‐mediated pathway (Figure 2e,f).

**FIGURE 2:**
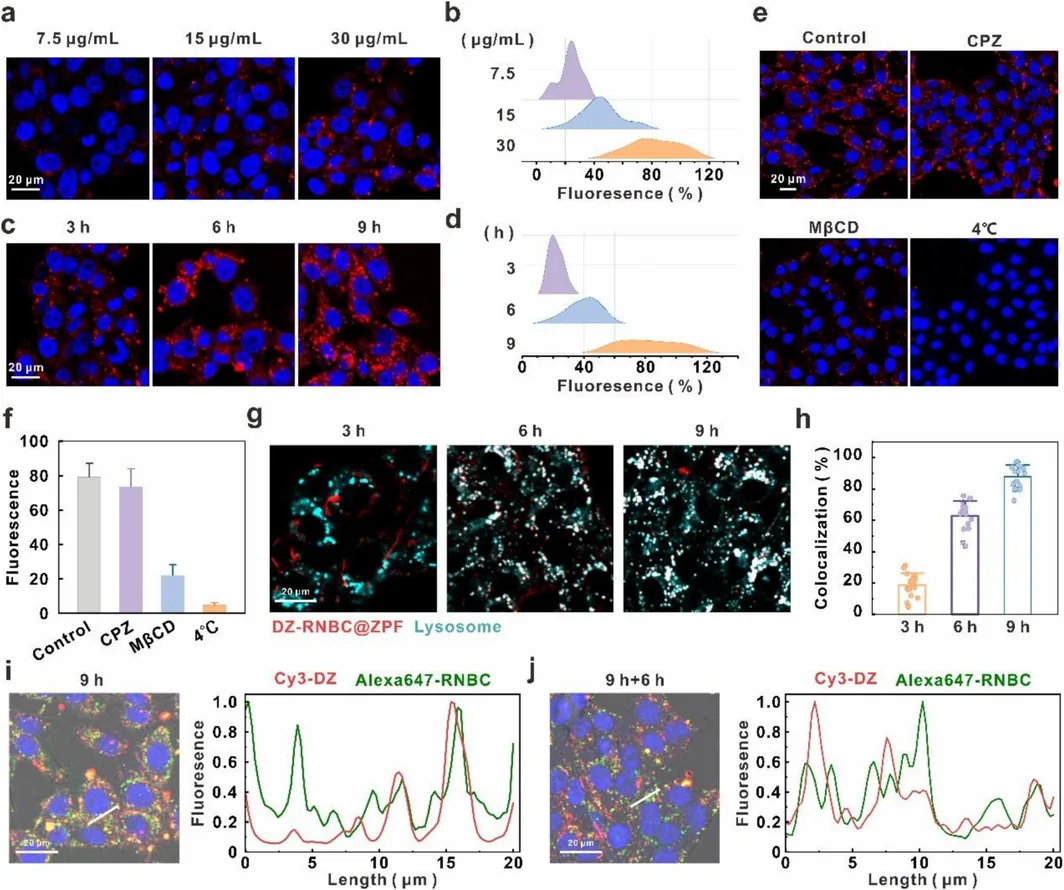
Spatiotemporal cellular uptake and intracellular processing of DZ‐RNBC@ZPF. (a and b) Concentration‐dependent cellular internalization: (a) Confocal laser scanning microscopy (CLSM) images and (b) quantitative fluorescence analysis of 4T1 cells incubated with DZ‐RNBC@ZPF (0–30 μg/mL) for 3 h. (c and d) Time‐course uptake kinetics: (c) CLSM images and (d) fluorescence intensity quantification showing accumulation over 9 h. (e and f) Endocytic pathway characterization: (e) CLSM images and (f) quantitative inhibition analysis following pretreatment with methyl‐β‐cyclodextrin (MβCD, caveolin inhibitor), chlorpromazine (CPZ, clathrin inhibitor), or incubation at 4°C (energy depletion). (g and h) Lysosomal trafficking: (g) CLSM images and (h) Quantification of colocalization (Manders' coefficient, %) coefficients demonstrating progressive colocalization (red: DZ‐RNBC@ZPF; green: Lysotracker) and subsequent escape (0–9 h). (i and j) Cargo dissociation analysis: Fluorescence line profiles of (i) immediately after 9 h uptake and (j) 6 h post‐removal, showing colocalized (yellow overlap) then separated signals (Cy3‐DZ: Red; Alexa 647‐RNBC: Green), confirming lysosomal escape and biomolecular dissociation. Scale bars: 20 μm (a, c, e, g, i, j).

After internalization, the nanoparticles trafficked to lysosomes. Co‐localization studies with LysoTracker Green showed high overlap (85%) at 9 h, confirming lysosomal accumulation (Figure 2g,h). To confirm payload release, we used dual‐fluorescence labelling (Cy3‐DZ and Alexa647‐RNBC). Line‐profile analysis showed initial co‐localization followed by significant spatial separation 6 h post‐removal, confirming acidic lysosomal‐triggered framework disassembly and cytosolic release (Figure 2i,j).

### 3.3 Intracellular Cascade Activation and Synergistic Cytotoxicity

The DZ‐RNBC@ZPF platform is designed to execute a sequential therapeutic cascade, effectively converting a genetic silencing event into a potent enzymatic storm. This strategy relies on a precise sequence of intracellular events, which we validated step‐by‐step (Figure 3a). First, lysosomal acidity degrades the ZPF, releasing the DZ and Zn^2+^ ions. These Zn^2+^ ions act as essential cofactors to activate the 8–17 DZ (Figure 3b). RT‐qPCR confirmed that DZ‐RNBC@ZPF treatment significantly knocked down GPX1 mRNA levels in 4T1 cells versus controls, verifying that the DZ retained its catalytic integrity and successfully engaged its target following intracellular delivery (Figure 3c).

**FIGURE 3:**
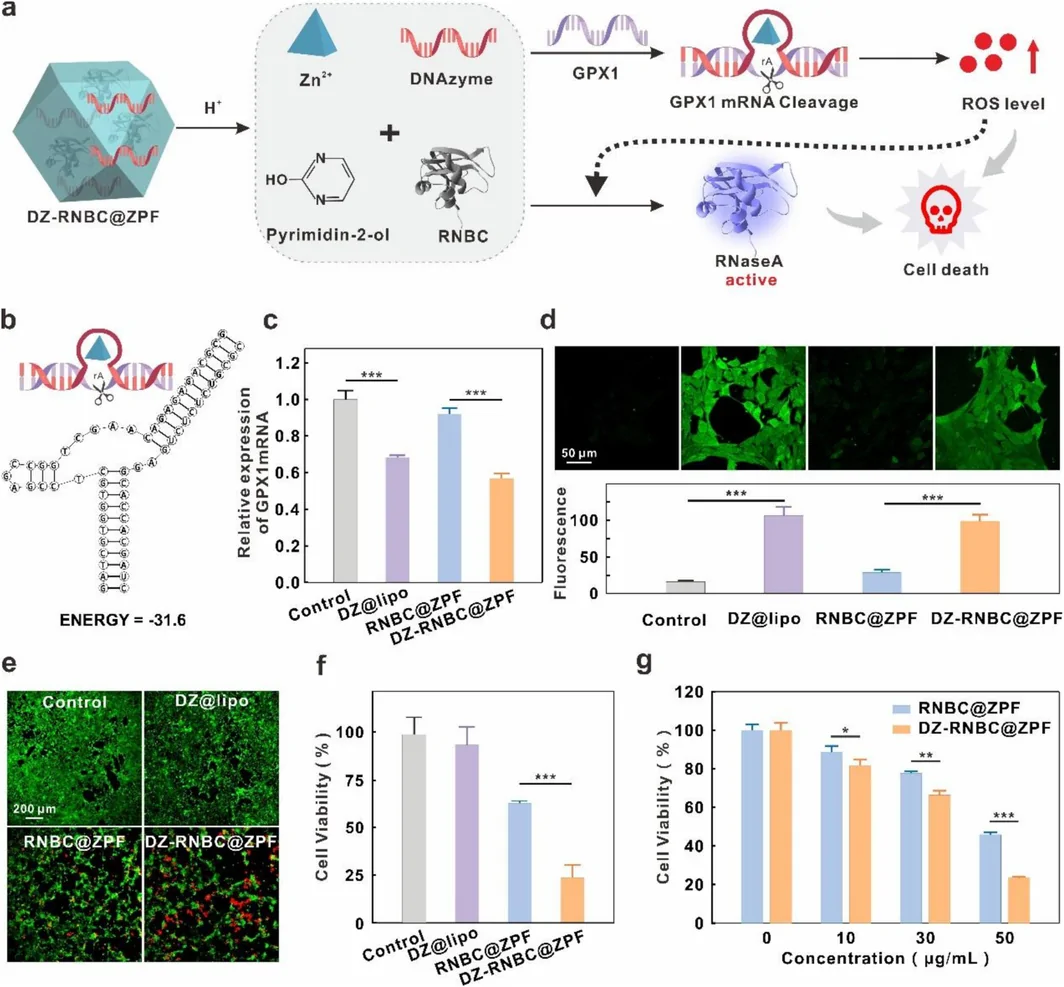
Intracellular cascade mechanism and synergistic anticancer activity of DZ‐RNBC@ZPF. (a) Therapeutic mechanism: Under acidic lysosomal conditions, DZ‐RNBC@ZPF dissociates to release Zn^2+^ (DNAzyme cofactor), RNBC prodrug, and GPX1‐targeting DNAzyme (DZ). DZ‐mediated GPX1 mRNA knockdown elevates intracellular ROS, triggering RNBC activation to catalytically active RNase A for apoptosis induction. (b) Secondary structure prediction (MFE: −31.6 kcal/mol) of the 8–17 DNAzyme with target GPX1 mRNA. (c) RT‐qPCR analysis of GPX1 mRNA treated with 30 μg/mL RNBC@ZPF, DZ‐RNBC@ZPF and equivalent Lipo‐delivered DZ for 24 h. (d) ROS amplification: CLSM images (top) and quantification (bottom) of DCFH‐DA fluorescence after different treatments. Scale bar: 50 μm. (e) Live/dead staining (Calcein‐AM/PI) and (f) quantitative analysis of 4T1 cells after 24 h incubation with 50 μg/mL RNBC@ZPF, DZ‐RNBC@ZPF or equivalent DZ@lipo. Scale bar: 200 μm. (g) MTT assay results showing dose‐dependent cytotoxicity of RNBC@ZPF and DZ‐RNBC@ZPF in 4T1 cells. *p < 0.05, **p < 0.01, ***p < 0.001 (*n* = 5).

The downregulation of Glutathione Peroxidase 1 (GPX1), a key antioxidant enzyme, disables the cell's primary defence against oxidative stress [39, 40]. We hypothesized that this genetic intervention would trigger a surge in intracellular ROS. Using the ROS probe DCFH‐DA, we observed a robust fluorescence increase in cells treated with DZ‐RNBC@ZPF, confirming the resultant ROS accumulation (Figure 3d). This induced oxidative stress serves as the trigger for the second phase of the cascade: the chemical activation of the protein prodrug. The engineered RNBC prodrug contains arylboronate caging groups designed to be cleaved specifically by elevated ROS. Upon cleavage, the steric blockade is removed, regenerating the native, catalytically active RNase A. This reactivation closes the loop, unleashing the enzyme to degrade cytosolic RNA and induce apoptosis.

The therapeutic potency of the synergistic cascade was evaluated. Live/dead staining (Calcein AM/PI) revealed extensive cell death in 4T1 cultures treated with the complete DZ‐RNBC@ZPF complex (50 μg/mL). In sharp contrast, treatments with the individual components—the DZ carrier (DZ@Lipo) caused minimal cytotoxicity; and the caged prodrug alone (RNBC@ZPF)—resulted in only moderate cytotoxicity (~30% cell death), which was significantly lower than that caused by the full cascade, confirming that neither oxidative stress nor the inert prodrug alone is sufficient for maximal lethality. Notably, the activated control, RNase A@ZPF, exhibited potent cytotoxicity at low concentrations (20 μg/mL), underscoring the critical need for the specific ROS‐trigger to unlock the prodrug's activity (Figures 3e,f, S9, S10). MTT assays quantified this synergy, showing cell viability dropped to ~23% at 50 μg/mL for the full complex, significantly outperforming treatments with individual agents (Figure 3g). Together, these results demonstrate that maximal cytotoxicity requires the sequential occurrence of acidic disassembly, DZ‐mediated ROS amplification, and prodrug activation.

### 3.4 In Vivo Antitumor Efficacy and Biosafety Evaluation

For systemic therapy, we cloaked the nanocomplex with 4T1 cell membranes. This biomimetic coating (DZ‐RNBC@ZPF@M) is designed to leverage homotypic adhesion for enhanced tumour retention and immune evasion (Figure S11) [41, 42, 43, 44, 45, 46]. The resulting DZ‐RNBC@ZPF@M nanoparticles exhibited a hydrodynamic diameter of approximately 231 nm (Figure S12a). The negative zeta potential further decreased after membrane coating, confirming successful encapsulation (Figure S12b). The nanomaterial demonstrated excellent colloidal stability over 7 days, as DLS monitoring revealed negligible changes in particle size or PDI (Figure S12c). We first evaluated the homologous targeting efficiency using CLSM. Alexa 647‐labelled DZ‐RNBC@ZPF (uncoated) and DZ‐RNBC@ZPF@M (membrane‐coated) were co‐incubated with NIH/3T3 cells and 4T1 cells for 6 h. Compared with the uncoated counterpart, the membrane‐coated nanocomplexes showed markedly higher accumulation in 4T1 cells (Figure S13a,d). Subsequently, the in vivo targeting efficiency of DZ‐RNBC@ZPF@M was examined by fluorescence imaging. In vivo fluorescence imaging 24 h post‐injection showed significantly higher tumour accumulation for the membrane‐coated complex versus the non‐coated version confirming targeting efficacy (Figure 4a,b).

**FIGURE 4:**
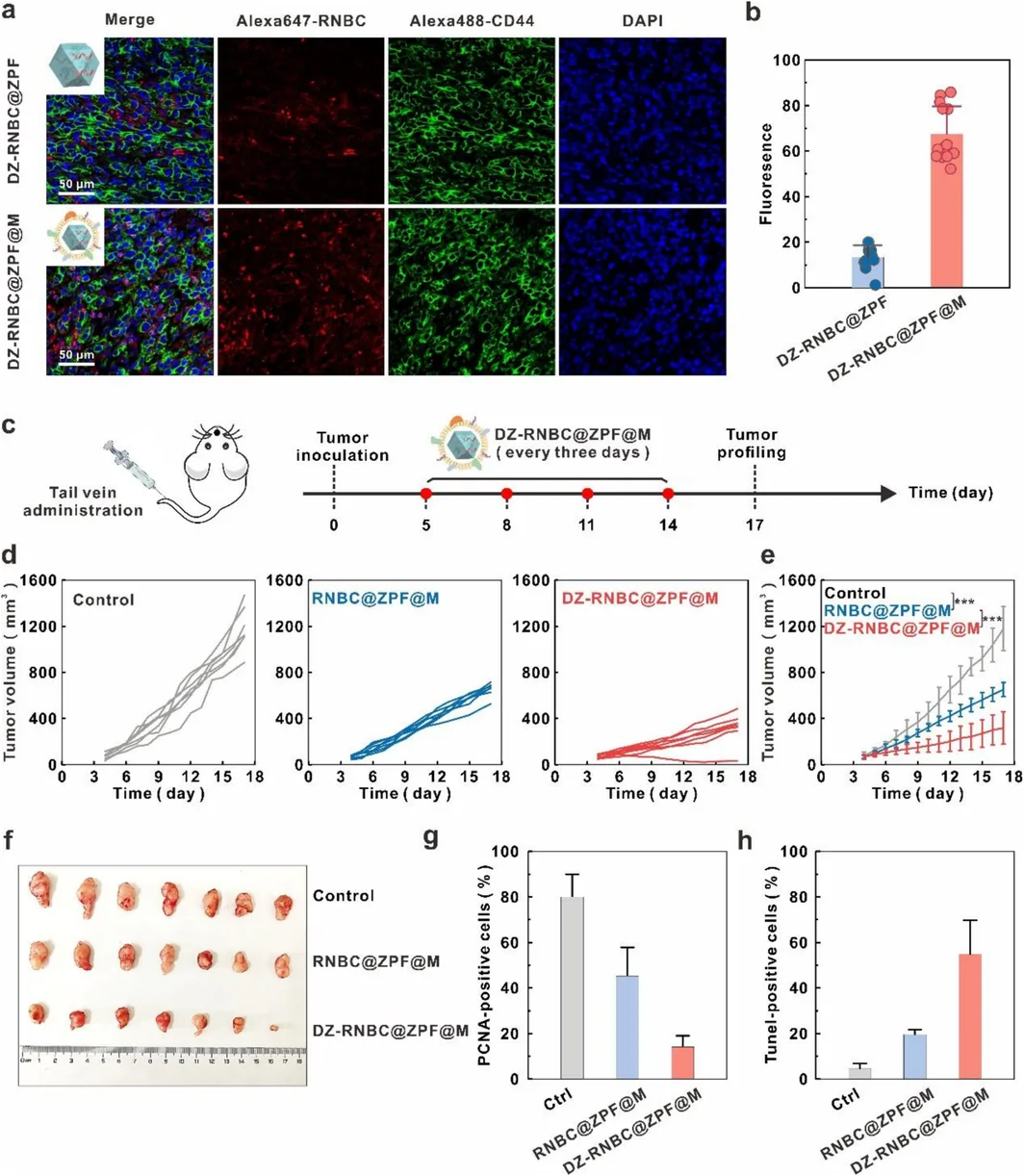
In vivo biodistribution and therapeutic efficacy of membrane‐camouflaged DZ‐RNBC@ZPF@M in a 4T1 breast cancer model. (a) Fluorescence imaging of tumour sections 24 h post‐injection shows retention of membrane‐coated DZ‐RNBC@ZPF@M and compared to uncoated nanoparticles. (b) Quantification reveals higher tumour accumulation for DZ‐RNBC@ZPF@M. Scale bar: 50 μm. (c) Treatment schedule (5 mg/kg RNBC equivalent, iv on days 5, 8, 11, and 14 post‐tumour inoculation). (d) Tumour volume growth curves for different treatment groups. (e) Statistical comparison of tumour volumes (*p < 0.05, **p < 0.01, ***p < 0.001). (f) Macroscopic tumour specimens at endpoint (day 17) exhibit pronounced size reduction in DZ‐RNBC@ZPF@M‐treated mice. (g) Immunohistochemical quantification shows PCNA‐positive proliferating cells. (h) TUNEL staining shows cell apoptosis.

The therapeutic efficacy was subsequently evaluated in an orthotopic 4T1 murine breast cancer model. Mice received intravenous injections of the various formulations every 3 days for a total of four doses (5 mg/kg equivalent RNBC) (Figure 4c). Monitoring of tumour kinetics revealed profound growth suppression in the DZ‐RNBC@ZPF@M group. By day 17, tumours in this group were the smallest, with significant regression compared to the PBS control and the RNBC@ZPF@M group (*p* < 0.001). Representative photographs of excised tumours visually confirmed this dramatic therapeutic effect (Figure 4d–f).

Histological analysis provided molecular evidence supporting the proposed mechanism of action in vivo. Immunohistochemical staining for PCNA showed a marked reduction in cell proliferation, while TdT‐mediated dUTP Nick‐End Labelling (TUNEL) assays revealed a high density of apoptotic cells in the DZ‐RNBC@ZPF@M group (Figures 4g,h, S14, S15). The observed antitumor effect is directly attributable to the designed intracellular cascade amplification mechanism. Specifically, upon acid‐triggered dissociation of the ZPF framework within lysosomes, the released Zn^2+^ ions activate the co‐encapsulated DZ, leading to efficient silencing of GPX1 expression. This genetic intervention disrupts the cellular antioxidant defence, resulting in a marked accumulation of ROS. The elevated ROS level not only inflicts direct oxidative damage but also serves as the critical trigger for cleaving the boronate cage of the RNBC prodrug, thereby restoring the catalytic activity of RNase A. Consequently, this self‐amplifying cascade potently induces apoptosis and suppresses tumour cell proliferation, as evidenced by the substantial increase in TUNEL‐positive cells (Figures 4h and S15) and the marked reduction in PCNA‐positive proliferating cells (Figures 4g and S14) in the DZ‐RNBC@ZPF@M treatment group. These histological markers align perfectly with our in vitro findings, confirming that the tumour regression was driven by the specific induction of apoptosis via the RNase A‐mediated cascade. The treatment showed excellent biosafety. Throughout the treatment course, no significant fluctuations in body weight were observed across any experimental groups, suggesting an absence of acute toxicity or cachexia (Figure S16). Comprehensive histological examination of major organs (heart, liver, spleen, lung, kidney) revealed no pathological lesions or signs of inflammation (Figure S17). Additionally, serum biochemical indicators for liver function (ALT, AST) and kidney function (BUN, Cr) remained well within physiological ranges (Figure S18). These data confirm the therapy's precision and the ZPF carrier's biocompatibility.

## 4 Conclusions

In this work, we present a bioresponsive nanoplatform (DZ‐RNBC@ZPF) that establishes a self‐sustaining therapeutic cascade, effectively bridging the divide between gene silencing and enzymatic therapy. Our design introduces three key innovations: First, we developed a redox‐amplification strategy that converts the typically insufficient tumour oxidative stress into a potent therapeutic signal. By coupling GPX1 mRNA cleavage (via Zn^2+^‐activated DZ) with ROS‐triggered RNase A activation, we created a positive feedback loop that both exploits and exacerbates tumour oxidative stress, effectively turning the tumour's metabolic vulnerability into a therapeutic strength. Second, the ZPF framework serves as a multifunctional carrier that simultaneously: (i) protects biomacromolecular payloads from degradation; (ii) provides essential Zn^2+^ cofactors for DZ activation; (iii) enables pH‐responsive payload release in lysosomes. Third, membrane camouflage confers tumour‐targeting specificity while evading immune clearance, as demonstrated by 4T1 tumour‐bearing mouse studies showing striking tumour suppression with no observable systemic toxicity (ALT/AST, BUN/Cr levels comparable to controls). This work establishes a new paradigm for smart therapeutics—where nanocarriers do not simply deliver drugs, but actively participate in therapeutic signal transduction. The modular design principles demonstrated here (protein‐structure‐directed ZPF, cell membrane camouflage, and biocatalytic cascades) provide a blueprint for developing next‐generation nanomedicines capable of precise molecular interventions. Future adaptations could incorporate additional diagnostic reporters or immune‐modulating components, further expanding the platform's therapeutic potential.

## Author Contributions

**Yan Huang:** data curation, formal analysis, investigation, writing – original draft. **Xingjie Hu:** conceptualisation, data curation, writing – review and editing. **Jinli Sun:** data curation, formal analysis, methodology. **Min Yin:** data curation, formal analysis, methodology. **Nan Chen:** conceptualisation, writing – review and editing, funding acquisition. All authors have given approval to the final version of the manuscript.

## Funding

The authors acknowledge the financial support from the Natural Science Foundation of Shanghai municipality (24ZR1455600), and National Natural Science Foundation of China (21974089).

## Conflicts of Interest

The authors declare no conflicts of interest.

## Supporting information


**Figure S1:** Synthesis and structural characterization of the nitrobenzyl carbamate (NBC) caging group. (a) Synthetic scheme of the ROS‐responsive NBC ligand from 4‐(hydroxymethyl)phenylboronic acid pinacol ester and p‐nitrophenyl chloroformate. (b) 1H NMR spectrum (500 MHz, CDCl_3_) of purified NBC showing characteristic signals, with peak assignments (a–f) corresponding to the chemical structure.
**Figure S2:** Biophysical characterization of the RNBC prodrug. (a) Coomassie‐stained 15% gel demonstrating successful RNBC synthesis, showing an increase in apparent molecular weight vs. native RNase A (13.7 kDa) consistent with conjugation of four NBC moieties. (b) Zeta potential measurements reveal charge neutralization from native RNase A to ‐RNBC in PBS (pH 7.4), confirming modification of surface lysine ε‐amines (****p < 0.0001, *n* = 5).
**Figure S3:** Functional validation of ROS‐responsive RNase A reactivation. (a) Agarose gel electrophoresis demonstrating RNA integrity after treatment with: (1) RNA ladder (1 kb), (2) Untreated RNA control, (3) RNA + native RNase A (positive control), (4) RNA + RNBC prodrug, (5) RNA + RNBC + H_2_O_2_, and (6) RNA + H_2_O_2_ (negative control).
**Figure S4:** In silico DNAzyme structural modelling. Predicted secondary structure of the 8–17 DNAzyme generated via RNA structure software, illustrating the catalytic core and target‐binding arms required for GPX1 mRNA recognition.
**Figure S5:** Protein quantification standard curve. BSA concentration standard curve obtained using the Coomassie Brilliant Blue method for the determination of protein loading capacity and release kinetics.
**Figure S6:**. Surface charge characterization of ZPF nanocomposites. Zeta potential measurements demonstrate successful functionalization of the zeolitic framework, with RNBC@ZPF acquiring greater negative charge upon DZ loading, consistent with incorporation of polyanionic DNAzymes.
**Figure S7:** Colloidal stability assessment of ZPF nanocomposites. (a and b) Hydrodynamic diameter and polydispersity index (PDI) monitoring of (a) RNBC@ZPF and (b) DZ‐RNBC@ZPF in deionized water (25°C) over 168 h, demonstrating excellent stability.
**Figure S8:** Morphological characterization of DZ‐RNBC@ZPF in acidic environment. SEM images at pH 7.4, and pH 5.0 after 30 and 60 min of incubation.
**Figure S9:** Cytotoxicity assessment of ZPF‐encapsulated RNase A. Dose–response of 4T1 cell viability after 24 h treatment with RNase A@ZPF (0–20 μg/mL) showing concentration‐dependent cytotoxicity.
**Figure S10:**. Therapeutic efficacy via live/dead cell imaging. Extended CLSM panels of 4T1 cells treated with DZ@Lipo, RNBC@ZPF, and DZ‐RNBC@ZPF, co‐stained with Calcein AM and PI to visualize the spatial extent of cell death. Scale bar = 200 μm.
**Figure S11:** Elemental characterization of membrane‐camouflaged nanoparticles. STEM‐EDS elemental mapping of DZ‐RNBC@ZPF@M showing uniform distribution of Zn, P, C, N, and O.
**Figure S12:** Colloidal stability assessment of DZ‐RNBC@ZPF@M nanocomposite. (a) Hydrodynamic diameter distribution (DLS) showing uniform nanoparticles. (b) Zeta potential measurements demonstrate successful fabrication of DZ‐RNBC@ZPF@M, the nanoparticles acquiring a greater negative charge upon cell membrane coating. (c) DZ‐RNBC@ZPF@M in deionized water (4°C) over 168 h, demonstrating excellent stability.
**Figure S13:** Membrane‐camouflaged DZ‐RNBC@ZPF@M promotes cellular uptake in 4T1 cells. (a) CLSM imaging of NIH/3T3 cells and 4T1 cells after incubation with DZ‐RNBC@ZPF and DZ‐RNBC@ZPF@M for 6 h showed significantly higher accumulation of the membrane‐coated DZ‐RNBC@ZPF@M in 4T1 cells compared with the uncoated nanoparticles. (b) Quantitative analysis of the intracellular fluorescence intensity in (a).
**Figure S14:** Histological evaluation of tumour cell proliferation. PCNA immunohistochemical staining of 4T1 tumour sections from treated mice, demonstrating significant reduction in proliferative activity in the cascade‐treated group.
**Figure S15:** Visualization of intratumoral apoptosis. Representative TUNEL‐stained tumour sections highlighting the induction of programmed cell death following treatment with DZ‐RNBC@ZPF@M.
**Figure S16:** Systemic safety monitoring. Body‐weight variation curves for 4T1 tumour‐bearing mice over the 14‐day treatment period, indicating no significant systemic toxicity or cachexia.
**Figure S17:** Systemic biosafety evaluation of DZ‐RNBC@ZPF@M. H&E‐stained tissue sections from major organs (heart, liver, spleen, lungs, kidneys) harvested 48 h after final treatment.
**Figure S18:** Haematological and biochemical safety profiling. Serum biochemistry analysis demonstrating preserved organ function following treatment with DZ‐RNBC@ZPF@M. Quantification of key biomarkers (ALT, AST, BUN, and Cr) shows no significant differences versus PBS control (*p* > 0.05, *n* = 7 mice/group).

## Data Availability

The data that support the findings of this study are available on request from the corresponding author. The data are not publicly available due to privacy or ethical restrictions.
