# Supplementary material for: A Zeolitic Pyrimidine Framework (ZPF) Nanoplatform Co‐Delivers a DNAzyme and a Protein Prodrug for Cascade‐Activated Tumour Therapy
Source: Cell Prolif. 2026 Apr 27:e70225. Online ahead of print. doi: 10.1111/cpr.70225 (PMC13325916; doi:10.1111/cpr.70225)
Supplement: Supplementary file 1 — Figure S1: Synthesis and structural characterization of the nitrobenzyl carbamate (NBC) caging group. (a) Synthetic scheme of the ROS‐responsive NBC ligand from 4‐(hydroxymethyl)phenylboronic acid pinacol ester and p‐nitrophenyl chloroformate. (b) 1H NMR spectrum (500 MHz, CDCl3) of purified NBC showing characteristic signals, with peak assignments (a–f) corresponding to the chemical structure. Figure S2: Biophysical characterization of the RNBC prodrug. (a) Coomassie‐stained 15% gel demonstrating successful RNBC synthesis, showing an increase in apparent molecular weight vs. native RNase A (13.7 kDa) consistent with conjugation of four NBC moieties. (b) Zeta potential measurements reveal charge neutralization from native RNase A to ‐RNBC in PBS (pH 7.4), confirming modification of surface lysine ε‐amines (****p < 0.0001, n = 5). Figure S3: Functional validation of ROS‐responsive RNase A reactivation. (a) Agarose gel electrophoresis demonstrating RNA integrity after treatment with: (1) RNA ladder (1 kb), (2) Untreated RNA control, (3) RNA + native RNase A (positive control), (4) RNA + RNBC prodrug, (5) RNA + RNBC + H2O2, and (6) RNA + H2O2 (negative control). Figure S4: In silico DNAzyme structural modelling. Predicted secondary structure of the 8–17 DNAzyme generated via RNA structure software, illustrating the catalytic core and target‐binding arms required for GPX1 mRNA recognition. Figure S5: Protein quantification standard curve. BSA concentration standard curve obtained using the Coomassie Brilliant Blue method for the determination of protein loading capacity and release kinetics. Figure S6:. Surface charge characterization of ZPF nanocomposites. Zeta potential measurements demonstrate successful functionalization of the zeolitic framework, with RNBC@ZPF acquiring greater negative charge upon DZ loading, consistent with incorporation of polyanionic DNAzymes. Figure S7: Colloidal stability assessment of ZPF nanocomposites. (a and b) Hydrodynamic diameter and p [file CPR-9999-e70225-s001.docx]

**Supporting Information**

**A Zeolitic Pyrimidine Framework (ZPF) Nanoplatform Co-Delivers a DNAzyme and a Protein Prodrug for Cascade-Activated Tumor Therapy**

Yan Huang^1, ‡^, Xingjie Hu^2, ‡^, Jinli Sun^2^, Min Yin^1^, Nan Chen^1,^*

^1^ Shanghai Engineering Research Center of Green Energy Chemical Engineering, Key Laboratory of Resource Chemistry of Ministry of Education, Shanghai Frontiers Science Center of Biomimetic Catalysis, College of Chemistry and Materials Science, Shanghai Normal University, 100 Guilin Rd., Shanghai 200234, P. R. China

^2^ State Key Laboratory of Oncogenes and Related Genes, Center for Single-Cell Omics, School of Public Health, Shanghai Jiao Tong University School of Medicine, Shanghai 200025 China

*Correspondence: nchen@shnu.edu.cn;

^‡^ These authors contributed equally to this work.


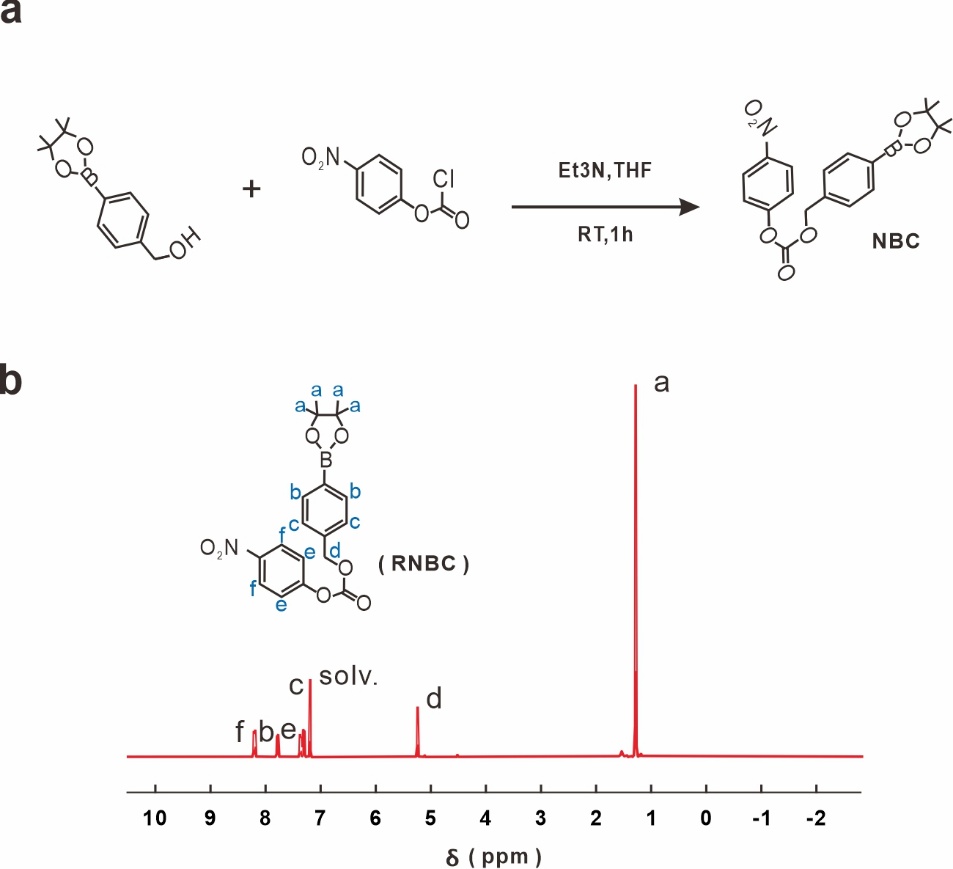


**Figure S1. Synthesis and structural characterization of the nitrobenzyl carbamate (NBC) caging group.** *(a)* Synthetic scheme of the ROS-responsive NBC ligand from 4-(hydroxymethyl)phenylboronic acid pinacol ester and p-nitrophenyl chloroformate. *(b)* 1H NMR spectrum (500 MHz, CDCl_3_) of purified NBC showing characteristic signals, with peak assignments (a–f) corresponding to the chemical structure.


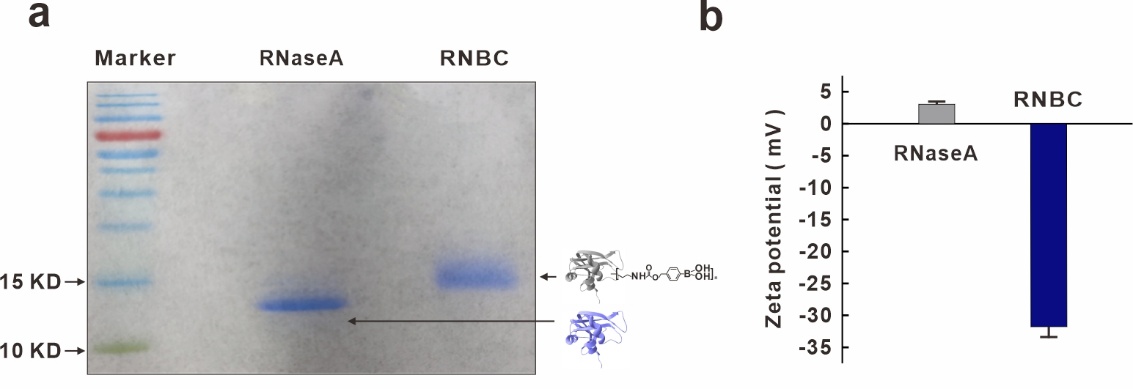


**Figure S2. Biophysical characterization of the RNBC prodrug.**
*(a)* Coomassie-stained 15% gel demonstrating successful RNBC synthesis, showing a increase in apparent molecular weight vs. native RNase A (13.7 kDa) consistent with conjugation of four NBC moieties. *(b)* Zeta potential measurements reveal charge neutralization from native RNase A to -RNBC in PBS (pH 7.4), confirming modification of surface lysine ε-amines (****p < 0.0001, n = 5).


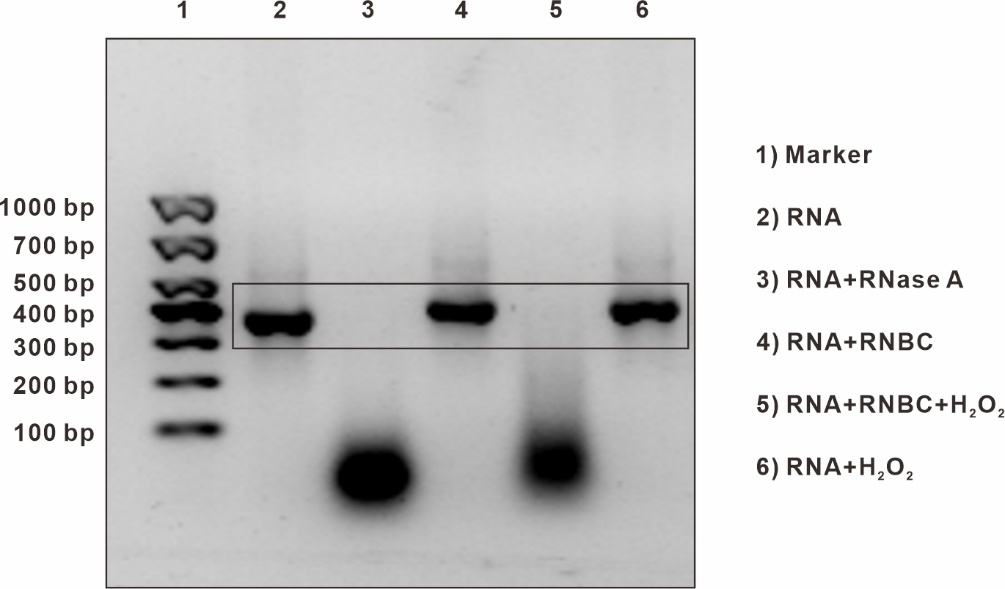


**Figure S3. Functional validation of ROS-responsive RNase A reactivation.**

*(a)* Agarose gel electrophoresis demonstrating RNA integrity after treatment with: (1) RNA ladder (1 kb), (2) Untreated RNA control, (3) RNA + native RNase A (positive control), (4) RNA + RNBC prodrug, (5) RNA + RNBC + H₂O₂, and (6) RNA + H₂O₂ (negative control).


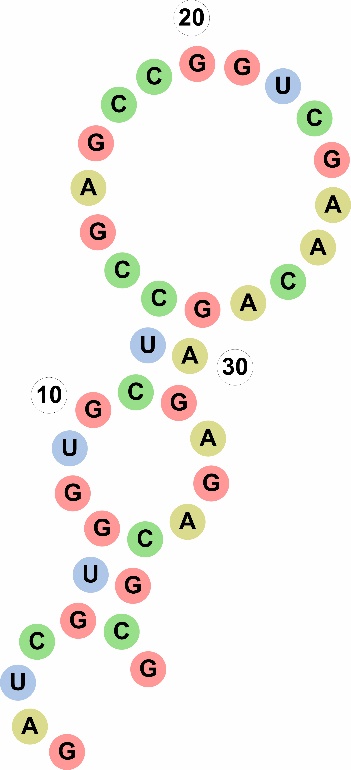


**Figure S4**. **In silico DNAzyme structural modeling**. Predicted secondary structure of the 8–17 DNAzyme generated via RNAstructure software, illustrating the catalytic core and target-binding arms required for GPX1 mRNA recognition.


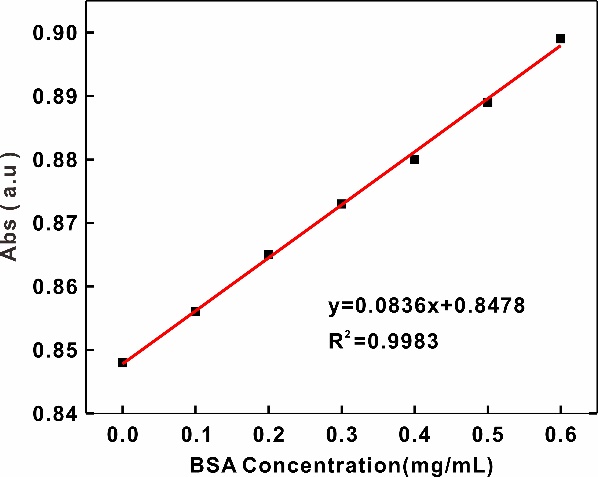


**Figure S5**. **Protein quantification standard curve**. BSA concentration standard curve obtained using the Coomassie Brilliant Blue method for the determination of protein loading capacity and release kinetics.


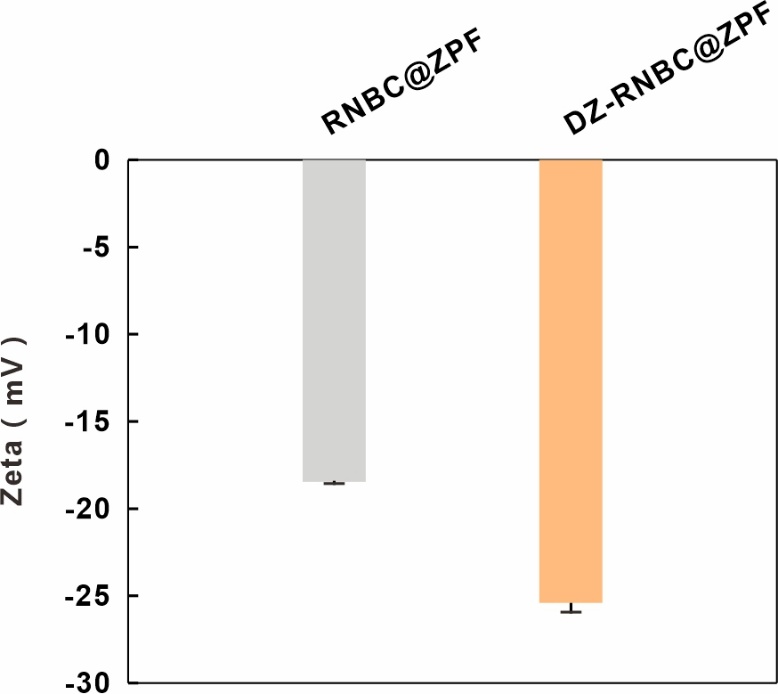


**Figure S6. Surface charge characterization of ZPF nanocomposites.** Zeta potential measurements demonstrate successful functionalization of the zeolitic framework, with RNBC@ZPF acquiring greater negative charge upon DZ loading, consistent with incorporation of polyanionic DNAzymes.


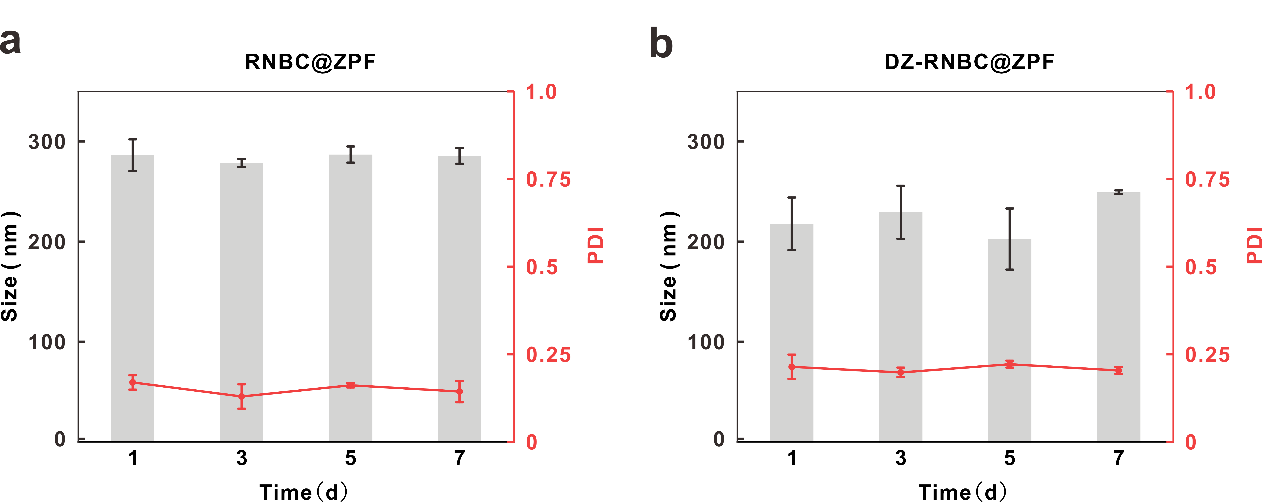


**Figure S7. Colloidal stability assessment of ZPF nanocomposites.** *(a-b)* Hydrodynamic diameter and polydispersity index (PDI) monitoring of (a) RNBC@ZPF and (b) DZ-RNBC@ZPF in deionized water (25°C) over 168 h, demonstrating excellent stability.


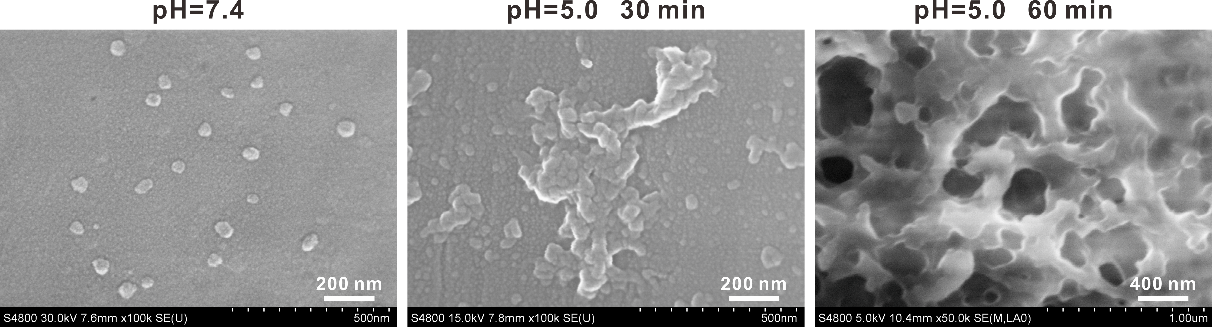


**Figure S8. Morphological characterization of DZ-RNBC@ZPF in acidic environment.** SEM images at pH 7.4, and pH 5.0 after 30 min and 60 min of incubation.


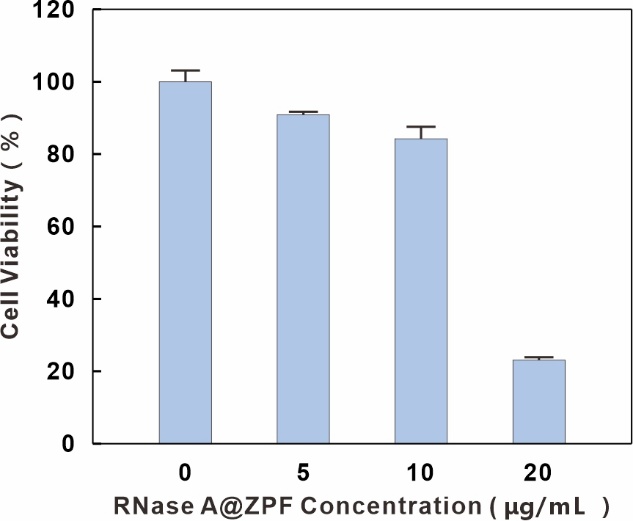


**Figure S9. Cytotoxicity assessment of ZPF-encapsulated RNase A.** Dose-response of 4T1 cell viability after 24 h treatment with RNase A@ZPF (0-20 μg/mL) showing concentration-dependent cytotoxicity.


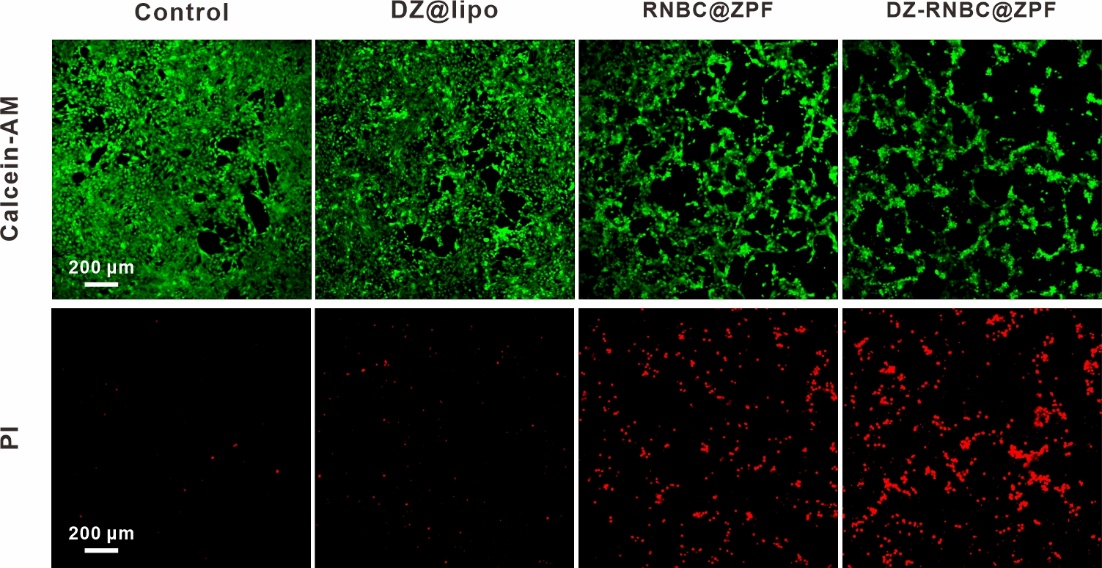


**Figure S10**. **Therapeutic efficacy via live/dead cell imaging**. Extended CLSM panels of 4T1 cells treated with DZ@Lipo, RNBC@ZPF, and DZ-RNBC@ZPF, co-stained with Calcein AM and PI to visualize the spatial extent of cell death. Scale bar = 200 μm.


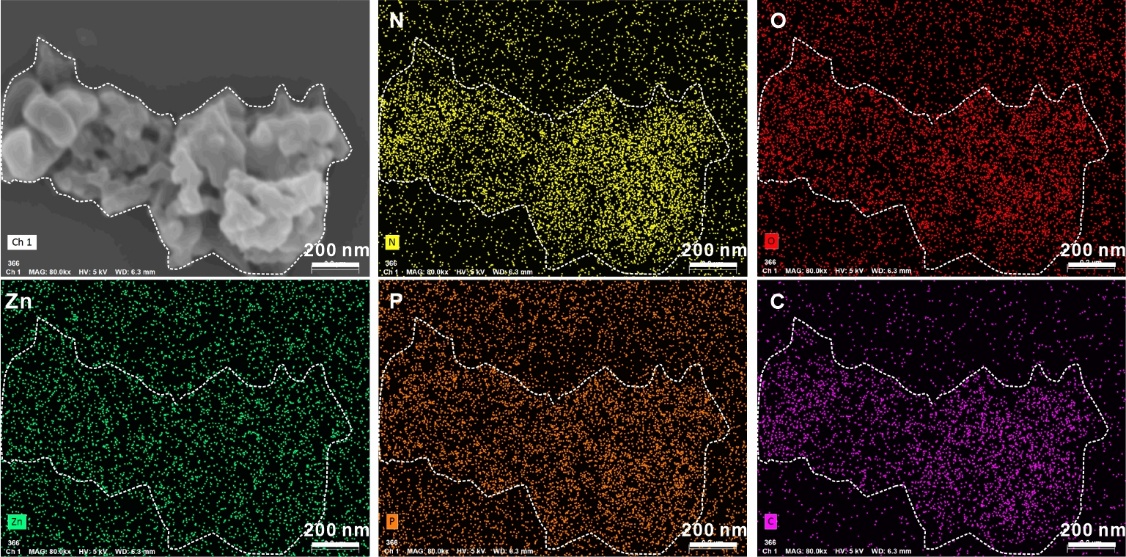


**Figure S11. Elemental characterization of membrane-camouflaged nanoparticles.** STEM-EDS elemental mapping of DZ-RNBC@ZPF@M showing uniform distribution of Zn, P, C, N, and O.


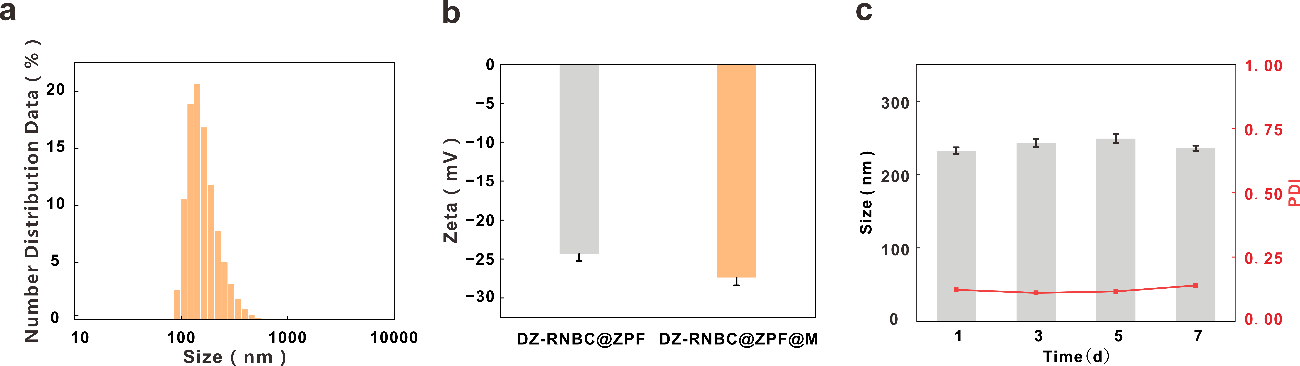


**Figure S12. Colloidal stability assessment of DZ-RNBC@ZPF@M nanocomposite.** *(a)* Hydrodynamic diameter distribution (DLS) showing uniform nanoparticles. *(b)* Zeta potential measurements demonstrate successful fabrication of DZ-RNBC@ZPF@M, the nanoparticles acquiring a greater negative charge upon cell membrane coating. *(c)* DZ-RNBC@ZPF@M in deionized water (4°C) over 168 h, demonstrating excellent stability.


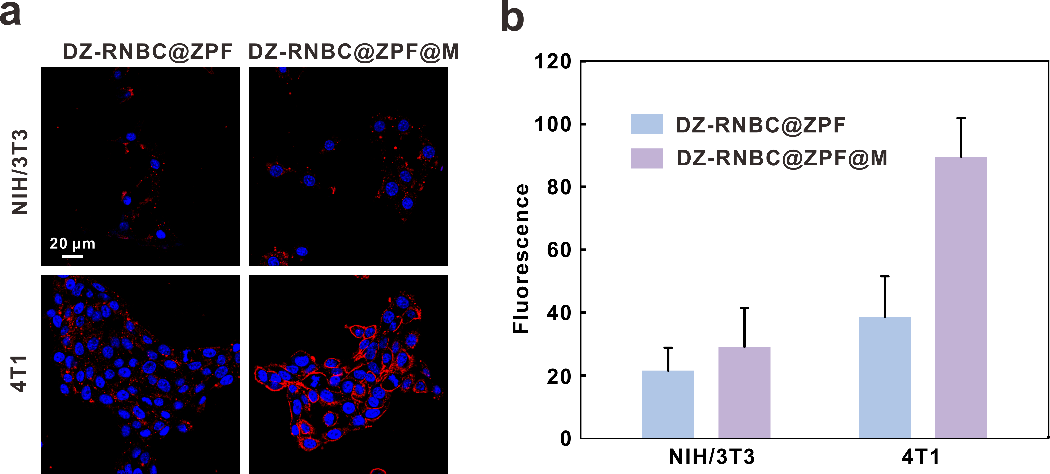


**Figure S13. Membrane-camouflaged DZ-RNBC@ZPF@M promotes cellular uptake in 4T1 cells.** *(a)* CLSM imaging of NIH/3T3 cells and 4T1 cells after incubation with DZ-RNBC@ZPF and DZ-RNBC@ZPF@M for 6 h showed significantly higher accumulation of the membrane-coated DZ-RNBC@ZPF@M in 4T1 cells compared with the uncoated nanoparticles. *(b)* Quantitative analysis of the intracellular fluorescence intensity in (a).


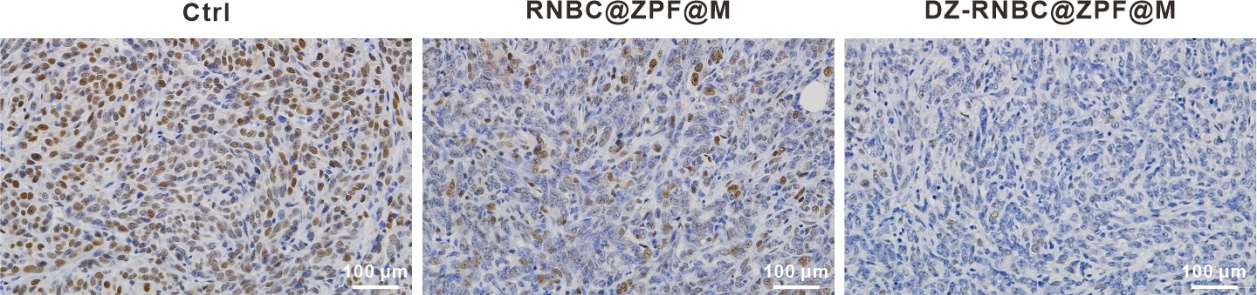


**Figure S14. Histological evaluation of tumor cell proliferation**. PCNA immunohistochemical staining of 4T1 tumor sections from treated mice, demonstrating significant reduction in proliferative activity in the cascade-treated group.


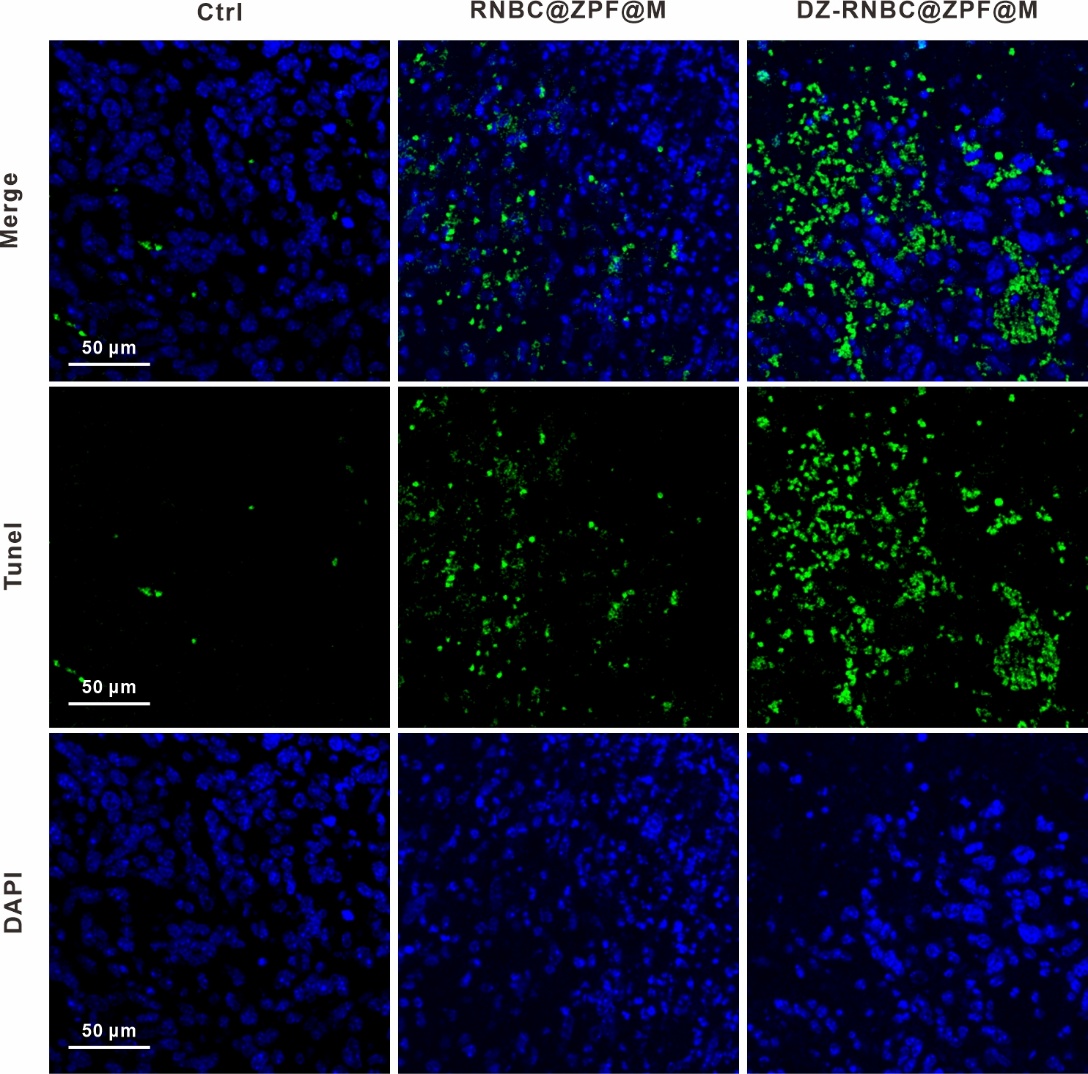


**Figure S15. Visualization of intratumoral apoptosis.** Representative TUNEL-stained tumor sections highlighting the induction of programmed cell death following treatment with DZ-RNBC@ZPF@M.


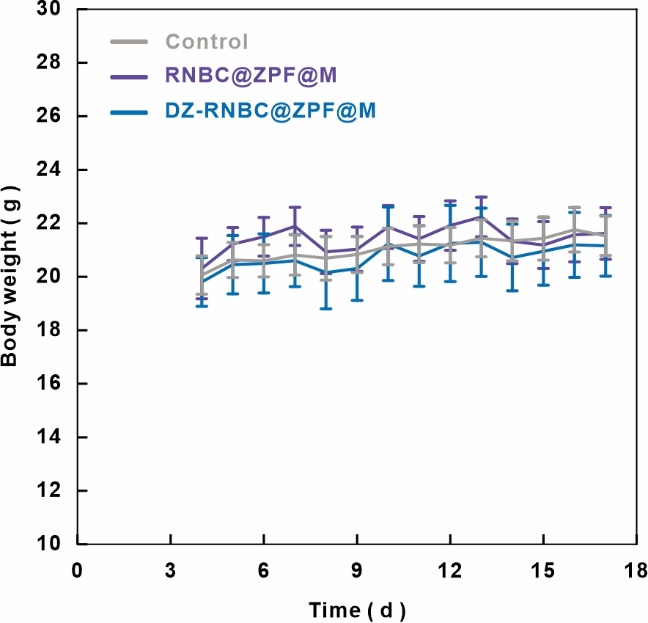


**Figure S16.** **Systemic safety monitoring.** Body-weight variation curves for 4T1 tumor-bearing mice over the 14-day treatment period, indicating no significant systemic toxicity or cachexia.


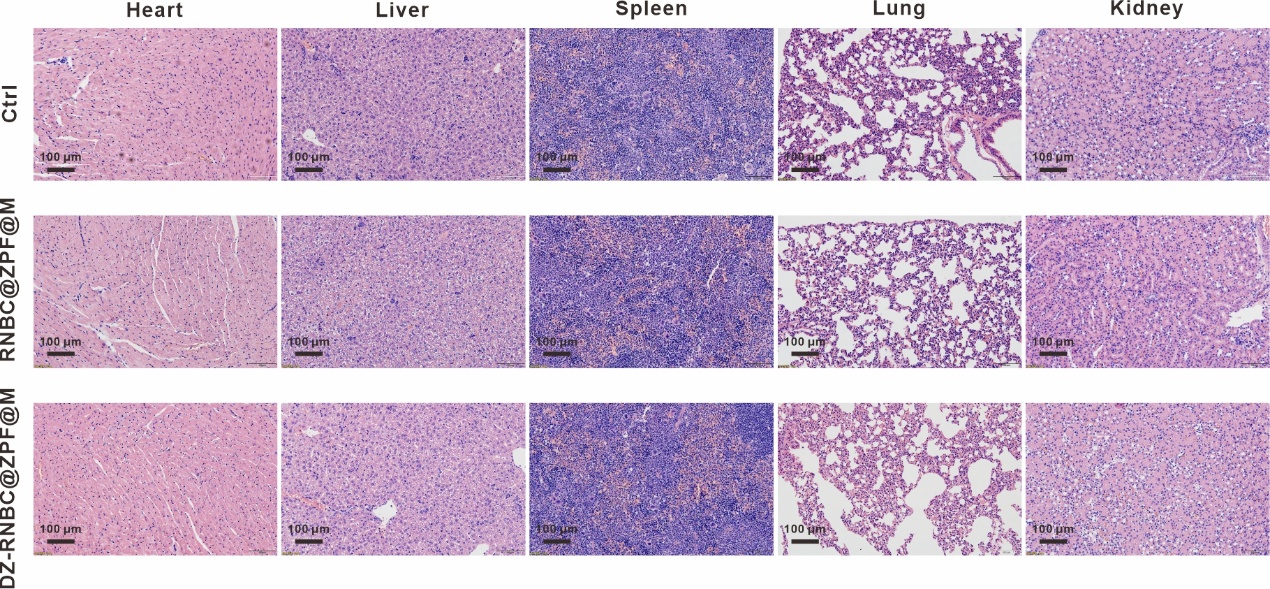


**Figure S17. Systemic biosafety evaluation of DZ-RNBC@ZPF@M.** H&E-stained tissue sections from major organs (heart, liver, spleen, lungs, kidneys) harvested 48 h after final treatment.


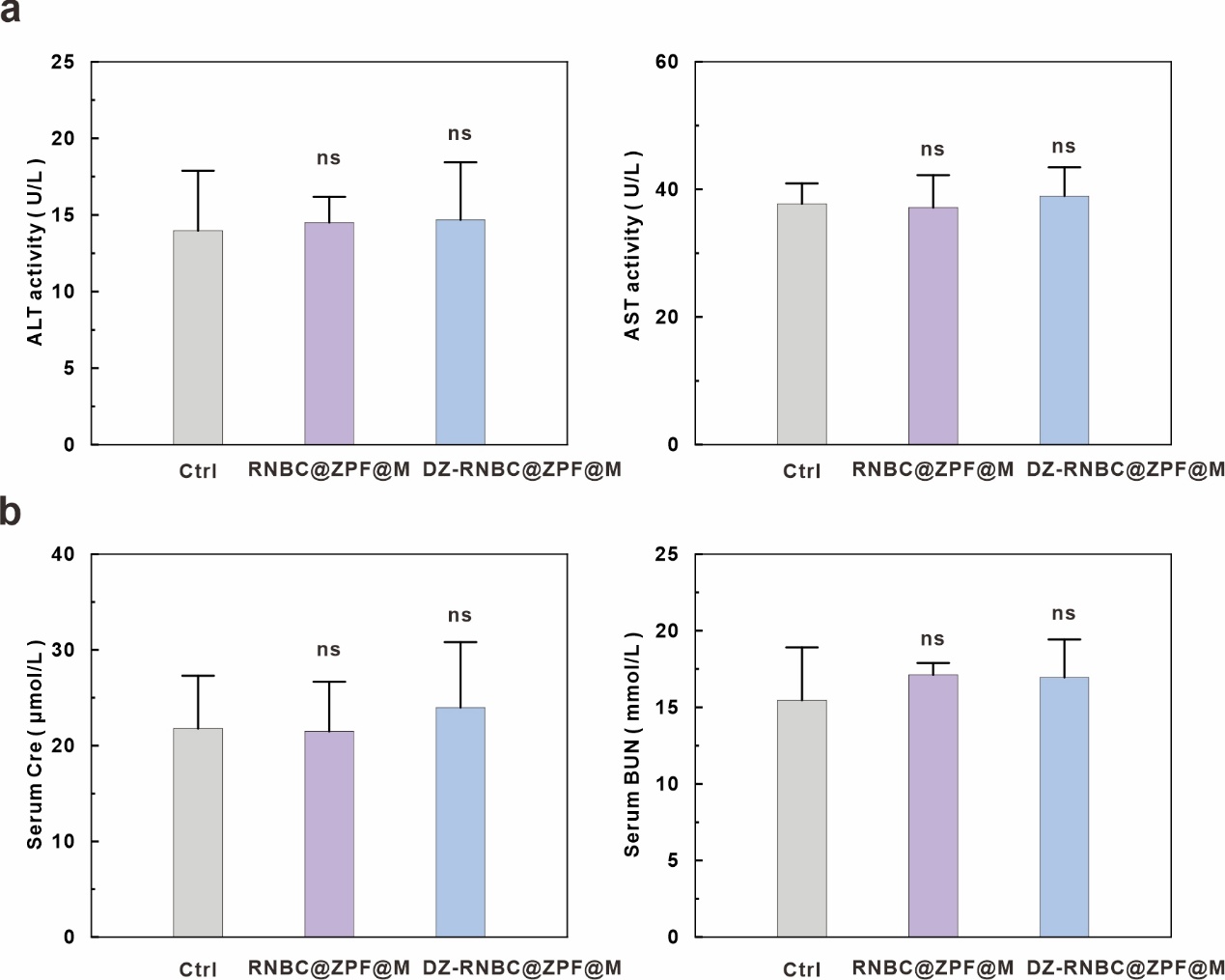


**Figure S18.** **Hematological and biochemical safety profiling.** Serum biochemistry analysis demonstrating preserved organ function following treatment with DZ-RNBC@ZPF@M. Quantification of key biomarkers (ALT, AST, BUN, and Cr) shows no significant differences versus PBS control (p > 0.05, n = 7 mice/group).
